# Early and Solid Protection Afforded by the Thiverval Vaccine Provides Novel Vaccination Alternatives Against Classical Swine Fever Virus

**DOI:** 10.3390/vaccines9050464

**Published:** 2021-05-06

**Authors:** Yaneysis Lamothe-Reyes, José Alejandro Bohórquez, Miaomiao Wang, Mònica Alberch, Marta Pérez-Simó, Rosa Rosell, Llilianne Ganges

**Affiliations:** 1 OIE Reference Laboratory for Classical Swine Fever, Institut de Recerca i Tecnologia Agroalimentàries, Centre de Recerca en Sanitat Animal IRTA-CReSA, 08193 Barcelona, Spain; yaneysis.lamothe@irta.cat (Y.L.-R.); josealejandro.bohorquez@irta.cat (J.A.B.); miaomiao.wang@irta.cat (M.W.); monica.alberch@irta.cat (M.A.); marta.perez@irta.cat (M.P.-S.); Rosa.rosell@irta.cat (R.R.); 2 Recombinant Biopharmaceuticals Laboratory, Pharmacology Department, School of Biological Sciences, Universidad de Concepción, 4030000 Concepción, Chile; 3 Departament d’Agricultura, Ramadería, Pesca i Alimentació (DARP), 08007 Generalitat de Catalunya, Spain

**Keywords:** CSFV, vaccination, vaccine, Thiverval strain, immune response, clinical protection, virological protection, challenge

## Abstract

Classical swine fever virus (CSFV) remains a challenge for the porcine industry. Inefficient vaccination programs in some endemic areas may have contributed to the emergence of low and moderate virulence CSFV variants. This work aimed to expand and update the information about the safety and efficacy of the CSFV Thiverval-strain vaccine. Two groups of pigs were vaccinated, and a contact and control groups were also included. Animals were challenged with a highly virulent CSFV strain at 21- or 5-days post vaccination (dpv). The vaccine induced rapid and strong IFN-α response, mainly in the 5-day immunized group, and no vaccine virus transmission was detected. Vaccinated pigs showed humoral response against CSFV E2 and E^rns^ glycoproteins, with neutralising activity, starting at 14 days post vaccination (dpv). Strong clinical protection was afforded in all the vaccinated pigs as early as 5 dpv. The vaccine controlled viral replication after challenge, showing efficient virological protection in the 21-day immunized pigs despite being housed with animals excreting high CSFV titres. These results demonstrate the high efficacy of the Thiverval strain against CSFV replication. Its early protection capacity makes it a useful alternative for emergency vaccination and a consistent tool for CSFV control worldwide.

## 1. Introduction

Classical swine fever (CSF) is a highly contagious disease that affects domestic pigs and wild boars. CSF poses a threat for the pig industry from sanitary and economic points of view, and its notification is mandatory to the World Health Organization for animal health (OIE) [1,2,3].

The CSF aetiological agent is the CSF virus (CSFV), an enveloped, single, positive-stranded RNA virus within the *Pestivirus* genus into the *Flaviviridae* family. The viral genome, of around 12.3 Kb of length, codifies for a unique polyprotein that after proteolytic processing yields mature non-structural and structural proteins [4,5]. Among the latter, E2 and E^rns^ glycoproteins are the main targets for neutralising antibodies [6]. 

Nowadays, the disease has been successfully eradicated in North America, Oceania, and a large part of the European Union [7]. However, despite the implementation of extensive eradication programs, CSF remains endemic in Asia, South and Central America, and the Caribbean [7]. The risk for CSFV re-emergence remains high, as shown by the recent outbreaks in countries including Korea, Colombia, Russia, Brazil, and even Japan, some of which had been CSF-free for decades [8,9].

Response policy to notification of an outbreak differs among different countries and depends on each particular situation [10]. The European Union followed a non-vaccination policy, accompanied by culling of infected animals and severe trade restrictions in the way to successful eradication of the disease. However, this approach carries significant ethical and economic implications. Meanwhile, in other countries where the virus remains endemic, vaccination poses a sustainable alternative to prevent massive losses and to contain the virus spread [2].

Modified live vaccines (MLVs) based on several attenuated virus strains, for example, C-strain, Thiverval, PAV-250, GPE-, and K-strain, are widely used [3,11]. The C-strain based vaccines are the most well studied ones, and their administration has proven to be safe and highly effective regardless of the genotype of the challenge strain [12]. However, CSFV persists in endemic countries even after active vaccination programs with these vaccines, mainly in areas that use either cell-culture or lapinized C-strain [2]. It is likely that this phenomenon is related to shortcomings in the vaccine program implementation rather than the effectiveness of the vaccines themselves [13]. Moreover, it has been hypothesized that this problem may have led to the emergence of new CSFV strains with low and moderate virulence that have been associated with chronic and persistent forms of CSF that hinder disease control [2].

The Thiverval vaccine, recommended in the OIE Manual for vaccination against CSFV [14], has shown high efficacy against the disease. However, the information available dates back to the 1970s and relies on techniques that are not up to date [15,16]. In addition, its capacity to confer early protection after single vaccination has not been evaluated.

The aim of the present study was to update the existing information on the efficacy of the Thiverval vaccine against CSFV at 21 days after a single inoculation, as well as its early protection capacity at five days after vaccination. The immune response generated after vaccination was evaluated. A contact group was included to assess the absence of vaccine virus transmission from the vaccinated animals. Likewise, the clinical, virological, and immunological protection afforded by the vaccine against a severe CSFV challenge was determined, using currently validated techniques.

## 2. Materials and Methods

### 2.1. Cells and Viruses

*Pestivirus*-free porcine kidney cell line PK-15 ATCC (CCL-33) cells were grown in Eagle’s minimum essential medium and supplemented with 5% fetal calf serum and used for viral production, titration, and neutralization assays. The commercially available Thiverval-strain vaccine (Coglapest^®^, CEVA, Santé Animale, France), (genotype 1.1) was used for vaccination in the in vivo assay. The highly virulent CSFV Margarita strain (genotype 1.4), which generates the lethal CSF form, was also employed [17]. The CSFV strain Alfort/187 was kindly provided by the CSFV EU Reference Laboratory (EURL), Hannover, Germany. The immune peroxidase monolayer assay (IPMA) with a swine polyclonal *Pestivirus* antibody was used to assess the viral replication [18], and viral titres were determined by endpoint dilution. The 50% tissue culture infective dose (TCID_50_) per millilitre was calculated using statistical methods previously described [19].

### 2.2. Experimental Design

Six-week-old piglets (*n* = 23), purchased from a commercial Spanish *Pestivirus*-free farm, were allocated in the animal biosafety level 3 (ABSL3) facility at IRTA-CReSA (Barcelona, Spain). Animals were numbered and divided randomly in four groups: groups A (pigs 1 to 7), B (pigs 8 to 13), C (pigs 14 to 18), and D (pigs 19 to 23). After the acclimatization period, group A was vaccinated with 2 mL (4 × 10^3^ plaque forming units (PFU)) of Thiverval strain through intramuscular injection in the neck, according to the manufacturer’s instructions. Group B pigs were inoculated with sterile PBS and were housed together with group A, serving as contacts to monitor the transmission capacity of the vaccine virus. Sixteen days after vaccination of group A, animals from group C were vaccinated with the Thiverval strain, following the same volume and dose of group A pigs. Finally, the group D were used as infection controls, being inoculated with PBS.

To evaluate the vaccine virus transmission and replication in tissues, at 21 dpv one animal from group A and two from group B were euthanized, and tonsil, thymus, spleen, and mesenteric lymph node samples were collected. At this time, at 21 dpv and 5 dpv for groups A and C, respectively, all the pigs from the four experimental groups were challenged by intramuscular injection in the right neck with 10^5^ TCID of CSFV Margarita strain. The animals were monitored, and rectal temperature was measured daily by a trained veterinarian starting on the day of vaccination and until the end of the trial. A clinical score from 0 to 6 was assigned according to the clinical status of the animals, as previously described [20,21]: 0, no signs; 1, mild fever; 2, mild to moderate clinical signs; 3, moderate clinical signs; 4, moderate to severe clinical signs; 5, severe clinical signs; and 6, death. Serum and nasal and rectal swabs were collected on the day of vaccination for all the groups and at 4, 7, and 14 dpv for groups A and B. Sampling was also carried out on the day of viral challenge and at 4, 10, and 13 days post-challenge (dpc) for all the experimental groups. Animals were euthanized before the end of the trial for animal welfare reasons when they exhibited prostration or reached a clinical score ≥5. At 13 dpc, the trial was ended and all surviving animals were euthanized and tonsil, spleen, and mesenteric lymph node were collected. Euthanasia was carried out by a pentobarbital overdose of 60–100 mg/kg of weight, administered via the jugular vein in accordance with European Directive. The experiment was conducted in accordance with the existing Spanish and European regulations and was approved by the Ethical Committee of the Generalitat de Catalonia, Spain under the animal experimentation project number 10908.

### 2.3. Determination of IFN-α Levels in Sera by ELISA Test

IFN-α concentration in sera was determined using a previously described in-house ELISA test [3,22] on the day of vaccination in all groups and at 4, 7, and 14 dpv for groups A and B. Serum samples were also evaluated on the day of CSFV challenge and at 4, 7, 10, and 13 dpc in the four experimental groups.

### 2.4. Evaluation of Humoral Response Against CSFV E2 and E^rns^ Glycoproteins and Neutralising Antibodies

CSFV E2 specific antibodies were determined in sera samples using a commercial ELISA kit (IDEXX Laboratories, Liebfeld, Switzerland). The blocking percentage values were determined following manufacturer’s instruction; values below 30% were considered as negative, between 30 and 40% were considered doubtful, and above 40% were considered as positive. Antibodies against the CSFV E^rns^ protein were evaluated using the pigtype© CSFV E^rns^ Ab test (Qiagen, Leipzig, Germany). Results were calculated by the S/P ratio (sample/positive control ratio). The S/P values below 0.3 were considered as negative, between 0.3 and 0.5 were doubtful, and above 0.5 samples were positive.

Furthermore, neutralising antibody response against the Margarita and Alfort/187 strains was determined by neutralization peroxidase linked assay (NPLA) [23]. Titres were expressed as the reciprocal dilution of serum that neutralised 100 TCID of 50% of the culture replicates.

### 2.5. Detection of CSFV RNA

Viral RNA was extracted from sera, nasal, and rectal swabs and tissue samples using IndiMag^®^ Pathogen Kit (Indical bioscience, Leipzig, Germany) according to the manufacturer’s instructions. Two RT-qPCR assays were used, one for the CSFV RNA detection [24] and the other for the specific CSFV Margarita strain [20]. Samples were considered negative when fluorescence was undetectable and positive when the threshold cycle (Ct) values were equal to or less than 40. The RNA load was defined as high (Ct value below 23), moderate (Ct values between 23 and 28), or low (Ct value above 28), as previously described [21].

### 2.6. Statistical Analysis 

The statistical analyses were performed using SPSS software, version 15.0 (SPSS Inc., Chicago, IL, USA), and “group” was set as the experimental unit. The non-parametric test (Kruskal–Wallis) was chosen to compare values obtained from the clinical scores; CSFV RNA in sera, swabs, and tissues; and IFN-α levels among all experimental groups. The significance level was set at *p* < 0.05 throughout the trial.

## 3. Results

### 3.1. Thiverval Vaccine Virus Has Weak Replication Capacity and Absence of Transmission among Pigs

**Figure 1:**
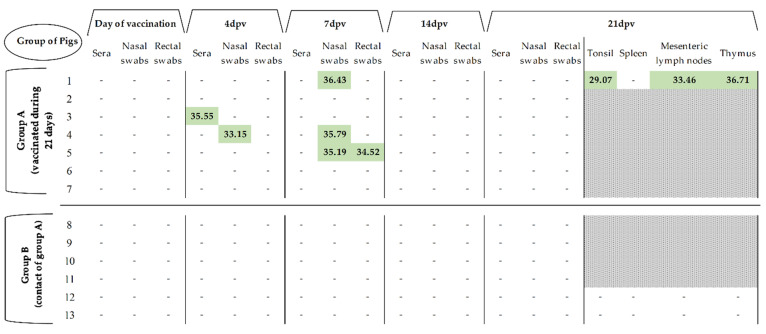
Detection of CSFV RNA load in samples and tissues during 21 days after vaccination. CSFV RNA load was detected by RT-qPCR and is expressed as Ct values. (-) symbol indicates samples in which fluorescence was not detected. Grey area indicates that the tissues were not collected at that time, in accordance with the experimental design described in the Materials and Methods section.

Thiverval strain RNA could not be detected in the majority of the samples during the 21 dpv (Figure 1). The vaccine virus was unable to generate viremia, shown by the general lack of viral RNA detection in sera, as only one animal showed low RNA load (Ct > 35) at four dpv. Likewise, low RNA load in nasal and rectal swabs was sporadically detected in one pig at 4 dpv and three at 7 dpv, with Ct values above 33. In addition, low or even absence of vaccine virus RNA load was also detected in the tissue samples from the euthanized vaccinated pigs at 21 dpv (Ct > 29), (Figure 1). During the 21 dpv, no vaccine virus RNA was detected in the contact group (group B) and all the animals from both groups were clinically healthy after vaccination.

### 3.2. A Single Vaccine Dose Conferred Early and Solid Protection Against CSFV Challenge at 5 and 21 dpv 

**Figure 2:**
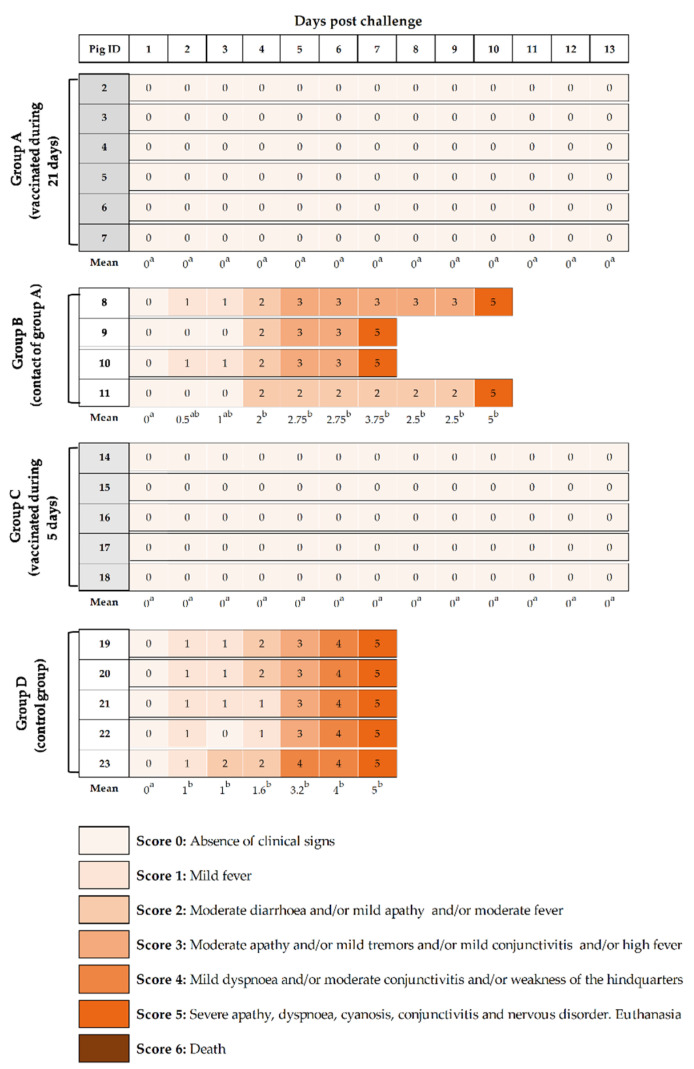
Clinical signs monitored after challenge. The individual clinical signs were recorded after CSFV “Margarita” strain infection. Animals 2 to 7 (group A) and 14 to 18 (group C) were challenged after 21 and 5 dpv, respectively. Animals 8 to 11 (group B), contacts for group A and 19 to 23 (group D), infection control group, were not vaccinated but challenged. Pigs were monitored daily for clinical signs during the 13 dpc or until euthanasia. Different shades of colour and the numerical clinical score represent the severity of the clinical signs as shown in the legend. Super-indexed letters in the mean clinical score value from each group are employed to represent statistically significant differences between the groups on that day; similar letters indicate no statistical difference, and different letters show statistical difference (*p* < 0.05).

Both vaccinated groups showed a total absence of clinical signs after challenge with a highly virulent CSFV strain with no statistic differences among them (*p* > 0.05). Thus, complete protection against CSFV challenge was afforded as early as 5 dpv with a single dose of the Thiverval strain. By contrast, non-vaccinated, control pigs (group D) started to show fever, leading to significantly higher clinical score values starting at 2 dpc (*p* < 0.05), which increased in the subsequent days, together with clinical signs compatible with severe acute CSF form (Figure 2). All pigs from this group developed apathy, conjunctivitis, weakness of the hindquarters, tremors, severe apathy, dyspnoea, and diarrhoea and had to be euthanized at 7 dpc due to animal welfare. Similar clinical signs were developed by non-vaccinated pigs from group B (contact of group A), which were statistically different from vaccinated groups (A and C) from day 4 until euthanasia (*p* < 0.05). These animals were euthanized from 7 to 10 dpc for animal welfare reasons (Figure 2).

### 3.3. The Thiverval-Strain Vaccine Strongly Activates the Innate Immunity in Pigs

**Figure 3:**
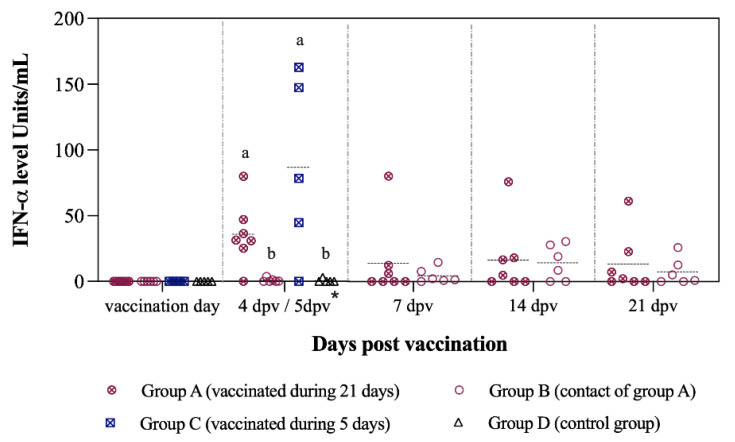
IFN-α levels in sera after vaccination during 21 days. The IFN-α levels were determined at different time points after vaccination with a single dose of the Thiverval-strain vaccine. Asterisk symbol (*) refers to 4 dpv for groups A and B, and 5 dpv for groups C and D. Letters above the symbols are used to represent statistically significant differences between the groups on that day: similar letters indicate no statistical difference and different letters show statistical difference (*p* < 0.05).

Induction of innate immunity, in terms of IFN-α levels in sera, was detected as early as 4 and 5 dpv with Thiverval strain in groups A and C, respectively. The IFN-α concentration was significantly higher in both vaccinated groups at this time-point (*p* < 0.05), reaching values of >150 units/mL in group C. Values of IFN-α in sera near to 0 units/mL were found in the majority of unvaccinated pigs from groups B and D (Figure 3).

### 3.4. Antibodies Against CSFV E2 and E^rns^ Glycoproteins with Neutralising Activity Are Detected Two Weeks after Vaccination

**Figure 4:**
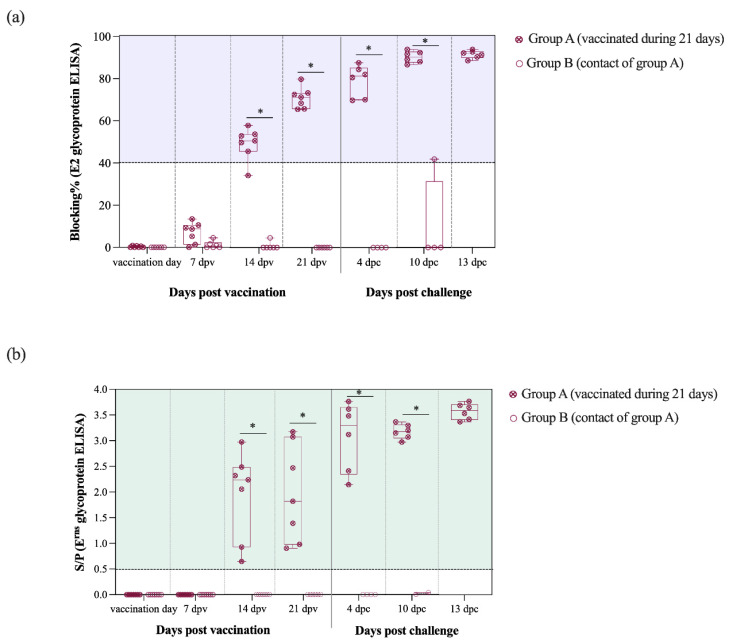
Kinetic of E2 and E^rns^ antibody response generated after vaccination and challenge for groups A and B. (**a**) Antibody response against the E2 glycoprotein was detected and is represented as blocking percentage. (**b**) Antibody response against E^rns^ was detected by ELISA and reported as the S/P value in accordance with the formula previously described. Statistically significant differences between the groups A and B in the antibody response against E2 (**a**) and E^rns^ (**b**) glycoproteins are represented by the asterisk symbol: (*) indicates *p* < 0.05.

E2-specific antibodies were detected at 14 dpv in 5 out of 6 vaccinated pigs from group A. Subsequently, at 21 dpv (day of CSFV challenge) the E2 antibody levels increased in all vaccinated pigs from group A and blocking percentage values were near 100% by the end of the trial. Antibody response against the E2 glycoprotein was statistically different between the groups A and B from the 14 dpv onwards (*p* < 0.05) (Figure 4a).

In the E^rns^ ELISA test, all group A pigs were positive starting at 14 dpv even though S/P values were highly variable between animals. After CSFV challenge, a sharp increase in the E^rns^ antibody levels was detected, with similar S/P values for all vaccinated pigs in group A (values > 3.5). From 14 dpv and until the end of the trial, the antibody response against E^rns^ glycoprotein was also significantly higher in the vaccinated pigs from group A than group B (*p* < 0.05) (Figure 4b).

**Figure 5:**
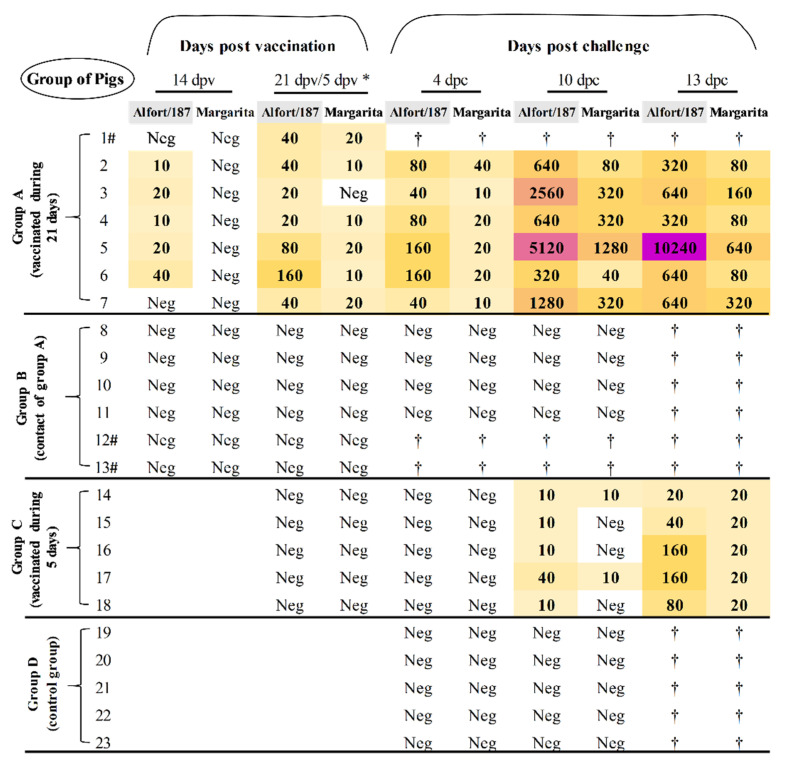
Determination of the neutralising antibody response during the trial. The neutralising antibody titres after vaccination and challenge were evaluated by NPLA. Neg: The sample was negative for NPLA test; † symbol indicates that the animal was euthanized before this time point; asterisk symbol (*) indicates 21 dpv for groups A and B, and 5 dpv for groups C and D. Symbol (#) indicates animals that were euthanized at the day of challenge, according to the experimental design, see Materials and methods section.

Accordingly, neutralising antibodies against Alfort/187 strain were detected starting at 14 dpv. From 21 dpv, titres were raised against Alfort/187 being above 1:30 in the majority of group A animals (Figure 5). After Margarita infection, a boost effect was observed in all vaccinated animals from group A, with titres as high as 1:640 at 13 dpc against the challenge strain. On the other hand, animals from groups B (contacts) and D (controls) elicited no neutralising antibody response at any time point during the study (Figure 5).

### 3.5. A single Thiverval Vaccine Dose Conferred Protection Against Viral Replication after CSFV Challenge at 21 dpv

**Figure 6:**
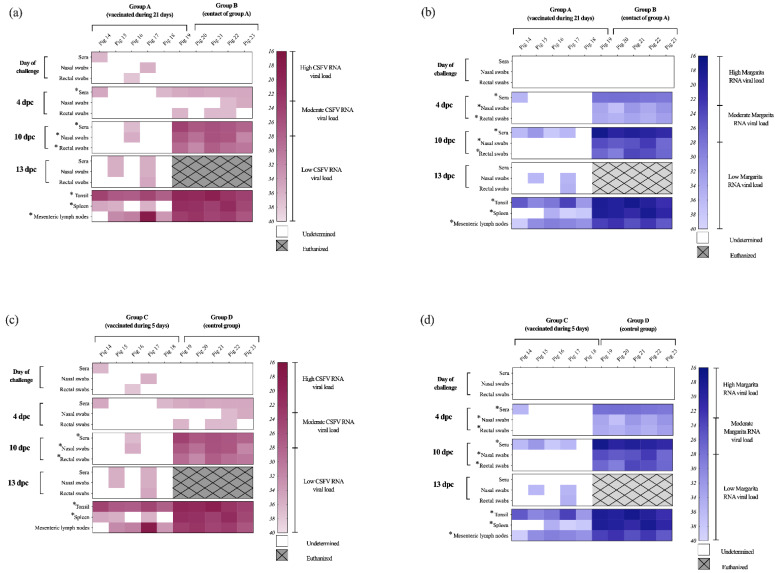
CSFV RNA detection at different time points after challenge in samples and tissues. The samples were analyzed by RT-qPCR for (**a**,**c**) the CSFV [24] and (**b**,**d**) Margarita strain RNA [20] viral load at different time points during the 13 dpc (or until euthanasia). White area indicates negative samples. Euthanized animals are represented in grey square with hatches. Asterisk symbol (*) indicates statistically significant differences of the Ct values between the groups (*p*  <  0.05).

Notably, after challenge, the absence of viral replication in blood was evidenced by the lack of CSFV RNA detection in sera samples from all vaccinated animals in group A (Figure 6a). Moreover, at 10 dpc a low RNA load was detected only in few rectal and nasal swab samples. However, no CSFV RNA was found in the remaining clinical samples analysed from vaccinated animals in group A (Figure 6a). Similarly, no viral RNA was found by either RT-qPCR assay in the spleen samples from group A (Figure 6a,b). Even though all the tonsil samples were positive for CSFV RNA, only 3 of them were positive by the Margarita-specific RT-qPCR with significantly lower RNA load (Ct value > 31) than the unvaccinated groups (*p* < 0.05). In the case of mesenteric lymph node, low viral RNA load was found in all the samples by the CSFV specific RT-qPCR (Figure 6a), while 4 out of 6 had Ct values corresponding with low RNA load by the Margarita-specific test (Figure 6b). Conversely, all the contact and control pigs (groups B and D) were positive in sera, nasal, and rectal swabs, after CSFV challenge. At 4 dpc, the Ct values corresponded with moderate and high RNA load by the Margarita-specific assay and were low by the CSFV RT-qPCR (Figure 6a,c). Between 7 and 10 dpc (time of euthanasia), the CSFV RNA load was significantly higher than the vaccinated pigs (*p* < 0.05), being mostly moderate by the CSFV RT-qPCR and high in the Margarita-specific test. All the tissue samples from these groups were positive by both assays, exhibiting mostly high RNA load that was statistically different (*p* < 0.05) to the vaccinated pigs (Figure 6 and Figure S1).

### 3.6. Thiverval Vaccine Generates Rapid CSFV Protection at Five Days after Vaccination in the Absence of Humoral Response

**Figure 7:**
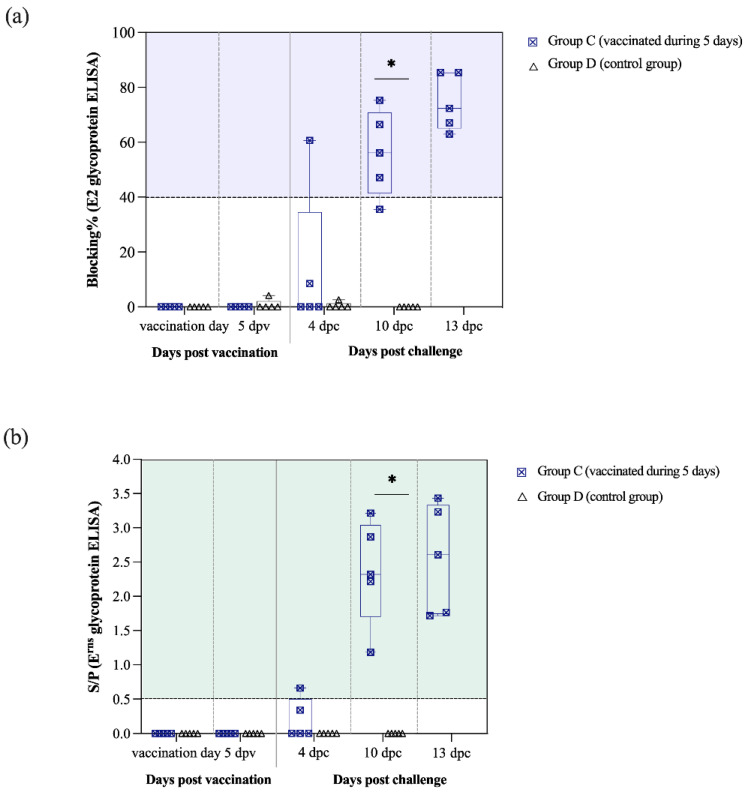
Kinetic of E2 and E^rns^ antibody response in pigs challenged at 5 dpv and controls (groups C and D). (**a**) Antibody response against the E2 glycoprotein was detected by ELISA and is represented as blocking percentage. (**b**) Antibody response against E^rns^ was detected by ELISA and reported as the S/P value in accordance with the formula previously described. Statistically significant differences between the groups C and D in the antibody response against the E2 (**a**) and E^rns^ (**b**) glycoproteins are represented by the asterisk symbol (*), indicating *p* < 0.05.

Pigs challenged at 5 dpv (group C) did not show antibody response in terms of E2 and E^rns^-specific antibodies or neutralising antibodies. Nevertheless, at 4 dpc, one pig from this group started to show antibodies against the E2 glycoprotein and two of them had antibodies to E^rns^ (Figure 7a,b). Afterwards, the number of group C animals with antibodies against both glycoproteins increased at 10 and 13 dpc, with all of them being positive at the end of the trial. Notably, the antibody response against the E2 and E^rns^ glycoproteins was statistically different between the groups C and D at 10 dpc (*p* < 0.05) (Figure 7a,b).

In terms of neutralising antibodies, titres were detected starting at 10 dpc, with all group C pigs being positive against Alfort/187 strain and two of them also showing low titres against Margarita. At 13 dpc, all the pigs from this group had neutralising antibodies against both viral strains analyzed, with titres being higher against Alfort/187 (between 1:20 and 1:160) than for Margarita (1:20) (Figure 5).

In direct contrast with the viral replication and excretion observed in the control animals (group D, described above), a complete lack of the CSFV challenge virus RNA was found in nasal and rectal swabs of the vaccinated pigs after challenge at 5dpv (group C), by the Margarita-specific test (Figure 6d and Figure S1). Moreover, viremia was nearly absent in animals from this group, as shown by the low RNA load detected in sera samples at 4 and 10 dpc. The Ct values were around 35.5 in the CSFV-specific test and ranged from 31 to 38 in the Margarita RT-qPCR. No Margarita RNA was detected in sera and swabs from group C at the end of the trial (13 dpc). Mostly low RNA load was found in the tissue samples from these pigs by the Margarita strain assay, while in the CSFV-specific test, all the tonsils showed moderate RNA load and the rest of the positive samples had low or absent RNA (Figure 6c,d and Figure S1).

### 3.7. CSFV-Induced Exacerbated Immune Response Is Prevented by the Thiverval Vaccine

**Figure 8:**
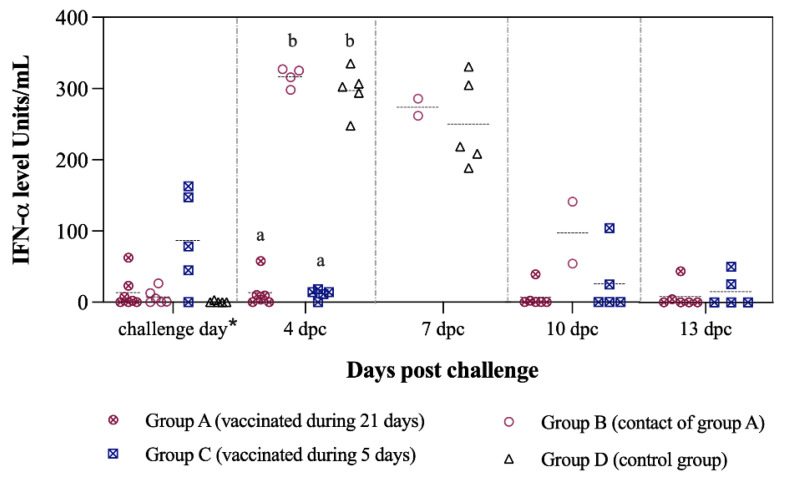
IFN-α levels in sera after challenge during 13 dpc. IFN-α levels were determined at different time points after challenge with CSFV “Margarita” strain. Asterisk symbol (*) refers to 21 dpv for group A and 5 dpv for group C. Letters above the symbols are used to represent statistically significant differences between the groups on that day: similar letters indicate no statistical difference, and different letters show statistical difference (*p* < 0.05).

After Margarita infection, significantly higher IFN-α levels (>300 units/mL) were found in sera from the non-vaccinated groups at 4 dpc (*p* < 0.05). In the control animals (group D), IFN-α concentration in sera remained high (>180 units/mL) at the time of euthanasia. Notably, the levels in vaccinated animals were near 0 units/mL (Figure 8).

## 4. Discussion

CSF is a re-emerging disease in swine, despite several years of intensive eradication programs. As has been explained, the inefficient vaccination programs in some endemic regions may have contributed to the emergence of new CSFV variants, putatively vaccine escape mutants, which threaten the epidemiological surveillance policies [13,25]. The reports of these proposed vaccine escape mutants have mostly come from areas where vaccination is performed with the C-strain. Notably, in some countries the production of this vaccine is still made in rabbits, mainly due to the poor replication of the C-strain in cells. This methodology remains controversial, not only because it constitutes a biosafety and ethical problem but also due to the inability to predict yield and scale up the production. Therefore, it is worth noting that other OIE-recommended vaccines, with stable, cell culture-based production systems, are available.

The present work aimed to expand and update the information about the efficacy, immune response, and transmission capacity of the CSFV Thiverval-strain vaccine virus in domestic pigs. In addition, its clinical and virological protection capacity after CSFV challenge with a highly virulent strain was evaluated at five and 21 days after single vaccination.

The results obtained here demonstrate that the vaccine virus is not transmitted from vaccinated to contact animals, even after 21 days of close contact in the same pen. This was shown by the absence of viral RNA and antibody response detection in the contact animals. This is further supported by the fact that this group developed the severe form of CSF after challenge, evidenced by the progressively worsening clinical signs, the rapid onset of the IFN-α response, and their inability to generate a CSFV specific antibody response.

Notably, a single dose of Thiverval vaccine can activate the immune response, as shown by the complete protection from clinical signs afforded in animals that were challenged either at 5 or 21 dpv, using a severe viral challenge, in accordance with OIE standards. Moreover, Thiverval vaccine primed the swine immune system to generate CSFV-specific antibodies against both the E2 and E^rns^ glycoproteins at 14 days after vaccination. These results are in line with previous findings reported for the C-strain vaccine and the modified CP7_E2alf vaccine candidate, another well-known alternative for CSFV vaccination [26]. The high levels of E2 and E^rns^ antibodies proved to have neutralising activity that correlated with robust virological protection detected in the vaccinated animals at 21 dpv. Notably, in addition to the clinical protection, all of the animals challenged at 21 dpv were protected from viremia, supporting the solid neutralising antibody protection conferred after vaccination. Moreover, the Thiverval strain avoided viral shedding by the vaccinated pigs at the end of the trial. Even though some animals were positive to CSFV RNA detection in swabs at 10 dpc, in all cases the RNA load was low and no virus was isolated in cell culture [21]. Furthermore, no viral replication was detected in spleen samples from vaccinated pigs, while in the case of tonsil and mesenteric lymph nodes, the Margarita strain RNA load detected was mostly low. Probably, the CSFV RNA load in these animals was a result of the prolonged exposure to a severe CSFV challenge, due to the close interaction between the vaccinated and contact animals that were secreting high amounts of virus. Considering that, the Thiverval strain was able to protect the animals not only from the initial challenge but also from the subsequent “double-challenge” posed by the contacts.

Remarkably, protection was achieved by the Thiverval strain as early as 5 dpv, even in the absence of antibody response. This suggests the strong activation of cellular immunity induced by the vaccine strain, which may have been able to control the highly virulent challenge. Likewise, the rapid and transient activation of innate immunity, in terms of IFN-α response against CSFV, may explain the solid protection capacity afforded by the Thiverval vaccine, as well as its efficacy to control viral replication shortly after vaccination. The antiviral and immunomodulatory effects of type I IFNs, such as IFN-α, have proven to be very important for impairing viral replication [27]. These results shed light into the mechanisms that underlie the vaccine protection against CSFV in the absence of specific humoral response. On the other hand, previous reports have shown that elevated levels of IFN-α are related to CSF disease severity [28]. In the present study, the vaccine strain protected the animals against the exacerbated IFN-α response, which was observed in the control animals. The Thiverval strain, continues to show novel applications, such as its capacity to protect as early as 5 dpv, that suggest it as an alternative for vaccination in emergency situations.

## 5. Conclusions

The Thiverval strain, an OIE-recommended vaccine that was first described decades ago, conferred solid clinical and virological protection, even against possible reinfection. Considering its efficacy and rapid protection capacity, this vaccine may be useful for endemic situations with continuous circulation of virus, as well as for vaccination in emergency situations. Thus, the Thiverval strain is an attractive vaccine for CSFV control worldwide, particularly in current endemic situations where new viral escape mutants may be circulating.

## Supplementary Materials

The following are available online at https://www.mdpi.com/article/10.3390/vaccines9050464/s1, Figure S1: Kinetic of CSFV RNA load detected in all samples from all experimental groups, collected after viral challenge.
