# Supplementary figures and images for: Early and Solid Protection Afforded by the Thiverval Vaccine Provides Novel Vaccination Alternatives Against Classical Swine Fever Virus
Source: Vaccines (Basel). 2021 May 6;9(5):464. doi: 10.3390/vaccines9050464 (PMC8148177; doi:10.3390/vaccines9050464)

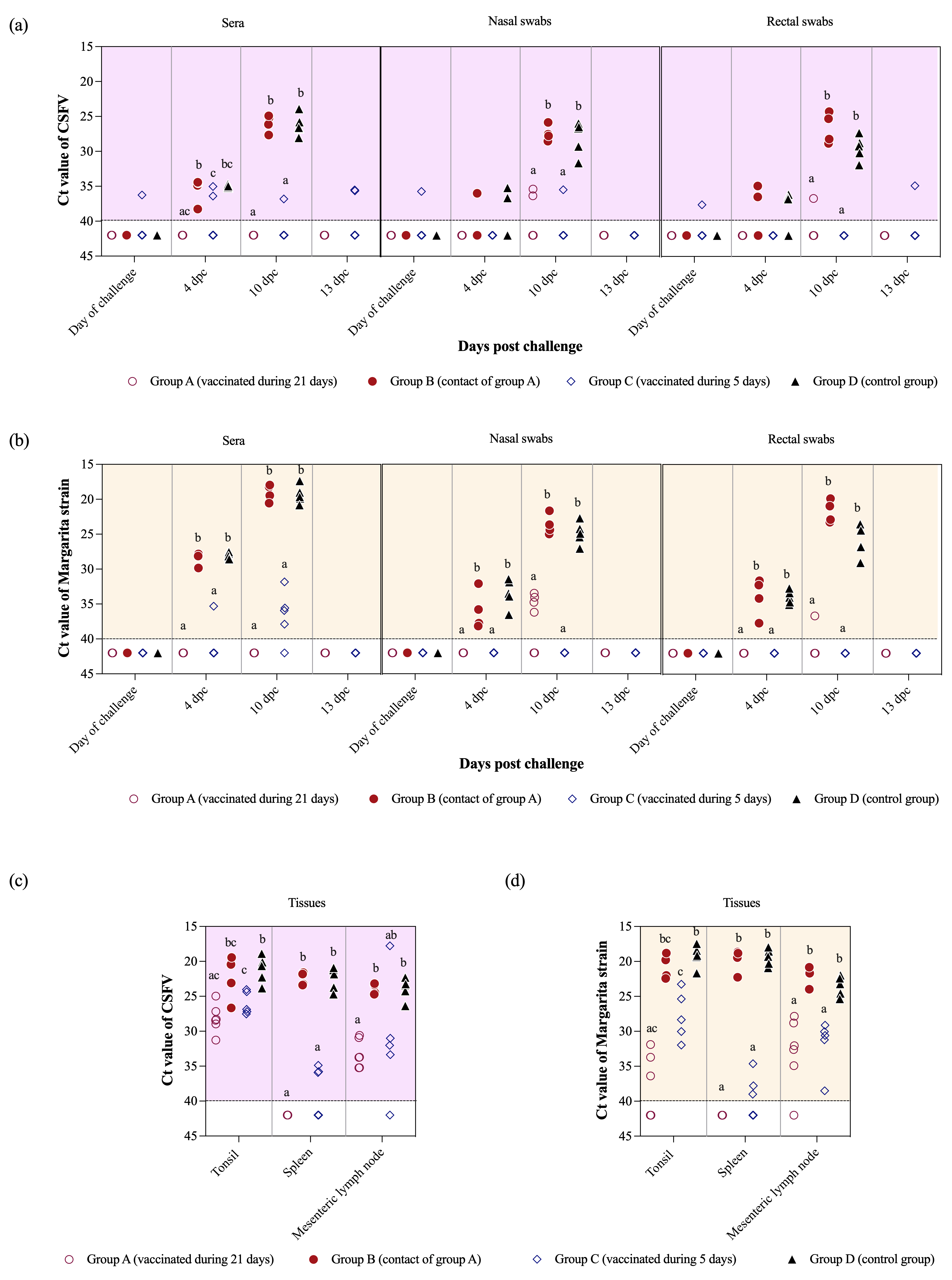

Supplement: Supplementary file 1 [file vaccines-09-00464-s001.zip › vaccines-1177774-supplementary.tiff]
